# Supplementary material for: Dry heat sterilization as a method to recycle N95 respirator masks: The importance of fit
Source: PLoS One. 2022 Jan 5;17(1):e0257963. doi: 10.1371/journal.pone.0257963 (PMC8730429; doi:10.1371/journal.pone.0257963)
Supplement: S1 File — (PDF) [file pone.0257963.s001.pdf]

S1 File. Fit Factor Assessment by Application of a Scanning Mobility Particle Sizer Spectrometer (SMPS).

Not every institution may have a TSI PortaCount Pro 8048 instrument available to conduct mask fit testing in times of crisis. We assessed if application of a Scanning Mobility Particle Sizer Spectrometer (SMPS) consisting of a condensation particle counter (CPC, TSI Inc. model 3775) and a differential mobility analyzer (DMA. TSI Inc., model 3080) allows to determine fit factors. The mask fit testing of N95 FFRs is based on detecting particles in a size range where the mask filtering material possesses a high filtration efficiency of greater than 99.98%. This is the case for 40 nm particles(23, 24). Application of a SMPS system allows size selection and particle count. Compared to the TSI PortaCount Pro 8048, a SMPS typically runs at lower sampling flow rates and as such has lower counting statistics. Consequently, one expects the SMPS system to yield larger uncertainties since the count uncertainty scales with the square root of the particle counts (e.g., 0.1 particles per cm^3 air is associated with ±316% error). Despite this disadvantage, we evaluated if a SMPS system could be employed to assess the mask fit, in times when TSI PortaCount Pro 8048 instrumentation is not available. We do not recommend using a SMPS system without further modification as a technique to conduct mask fit testing on a regular basis. For this a TSI PortaCount Pro 8048 or similar devoted instrumentation should be employed.

The SMPS was used to size-select 40 nm particles. The number of 40 nm particles in the room air and in the respirator during the above-mentioned exercises were measured and the fit factor derived. The aerosol and sheath flow of the DMA were 0.3 and 3 liter per minute (LPM), respectively. The aerosol measurement was corrected for diffusional losses in the tubing and the presence of multiple charged particles was accounted for. Since the sampling flow rate is slower than for the TSI PortaCount Pro 8048, the time period for each exercise was extended up to 5 minutes where the particle counts for the last 2 to 3 minutes were used for derivation of fit factors.

Supplemental figure 3 shows the results of conducting the mask fit testing procedure with a standard SMPS system. We derived the fit factor according to the equation as defined in NIOSH 42 CFR 84 (see Methods Section). Upper and lower bounds of the fit factor assumed the most conservative count estimates applying measured counts and their corresponding count error. Most conservative signifies, e.g., the greatest number of 40 nm particle in room air (including count uncertainty) over lowest number of 40 nm particles in respirator (subtracting count uncertainty). Supplemental Figure 3 clearly demonstrates that masks that failed the fit test using the TSI PortaCount Pro 8048, also failed the fit test in this setup. Interestingly, the Bacou Willson 801 autoclaved mask displays the lowest fit factor similar to the results shown in Fig. 3. The 3M 1860 untreated mask yields a fit factor of 95, close to a value of 100 for passing, with an upper bound of over 400. Since this mask efficiently filtered particles and had a very good fit on the person's face, the number of particles counted are small with the result, as mentioned above, of larger uncertainties. Within fit factor uncertainties, one may argue that the 3M 1860 untreated mask passed the fit test. However, considering the very different fit factor compared to the failed fit tested masks, may suggest that the SMPS system is more suitable to detect masks that fail the fit test.
