# Supplementary figures and images for: Dry heat sterilization as a method to recycle N95 respirator masks: The importance of fit
Source: PLoS One. 2022 Jan 5;17(1):e0257963. doi: 10.1371/journal.pone.0257963 (PMC8730429; doi:10.1371/journal.pone.0257963)

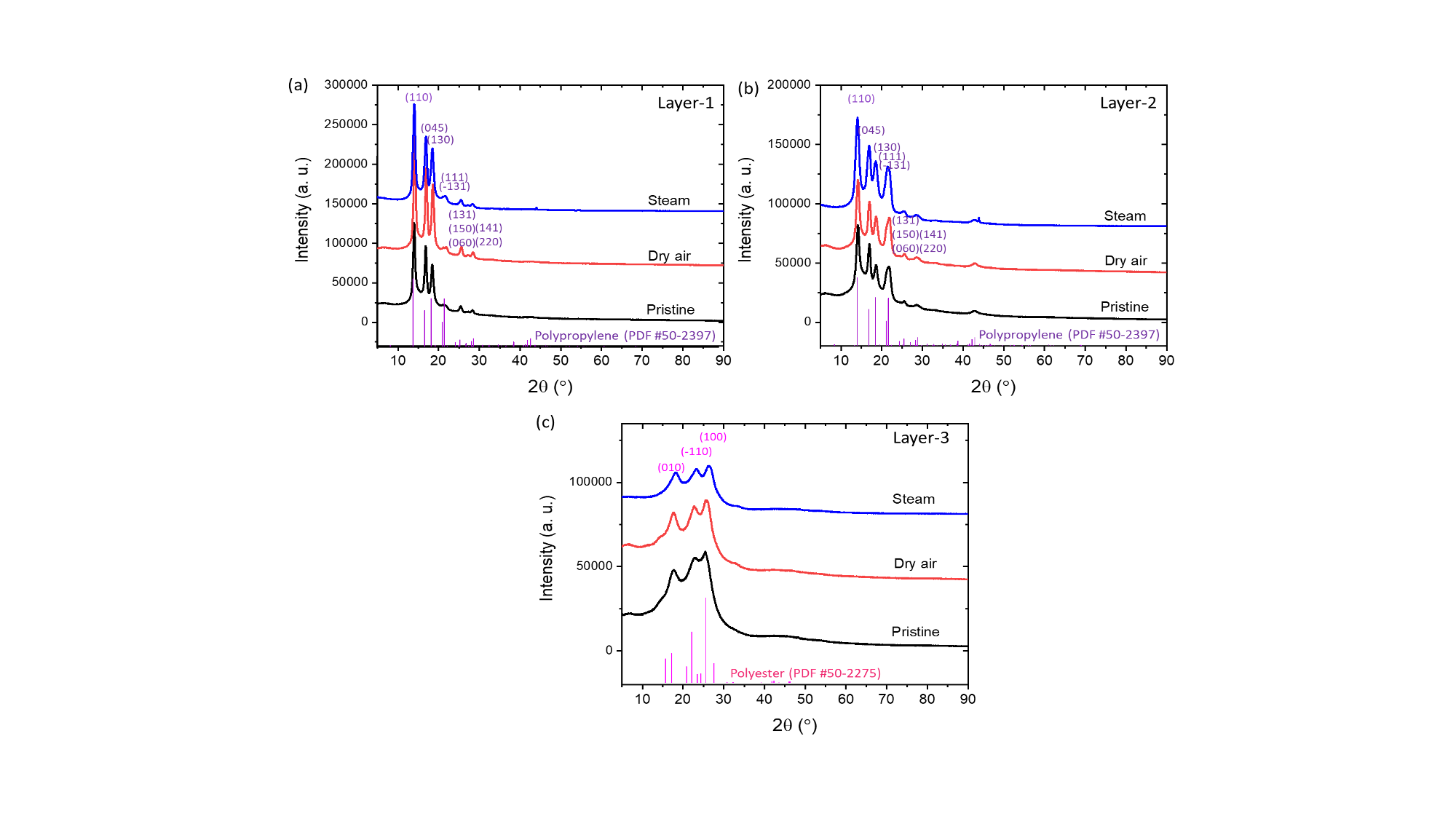

Supplement: S1 Fig — Compared with the XRD patterns of the pristine masks (black), after dry air treatment (red) and autoclave/steam treatment (blue), the 3M 1860 N95 FFR materials showed no compositional changes and insignificant crystallite sizes changes after both types of thermal treatment. Specifically, the respective crystallite sizes of the pristine, dry air treated and steam treated layer are 15, 17 and 14 nm for layer 1 (S1a Fig); and 11, 11 and 8 nm for layer 2 (S1b Fig). This indicated that dry air treatment slightly increased the crystallize size at layer 1 with no significant change at layer 2. Interestingly, steam treatment decreased crystallite size for both layers 1 and 2 for the 3M 1860 N95 FFR materials. No crystallite size was calculated for layer 3 due to its more amorphous character with significant peak overlap, and no obvious change was observed on layer-3 between the pristine and heat-treated samples (S1c Fig). (TIFF) [file pone.0257963.s002.tiff]

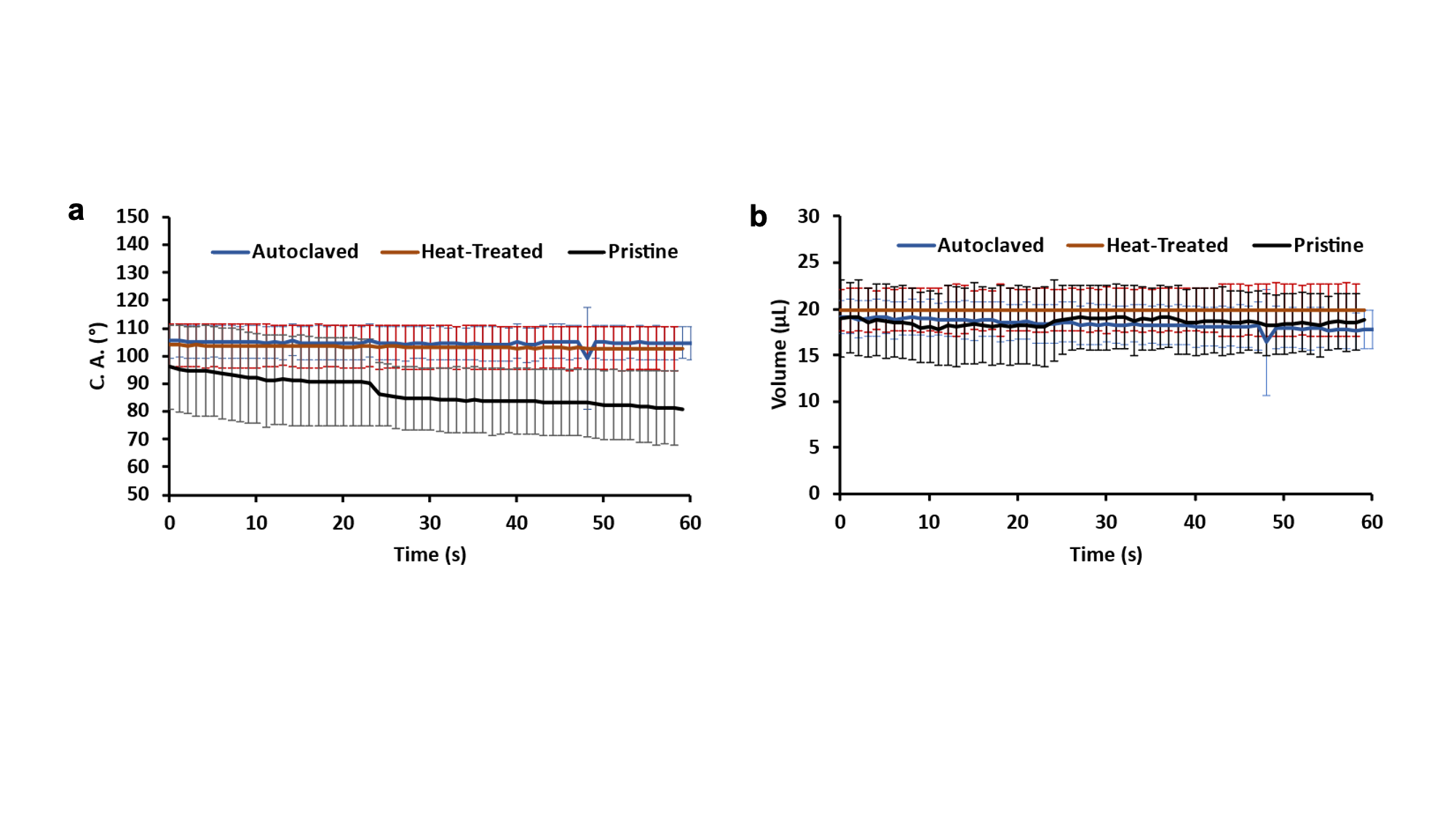

Supplement: S2 Fig — In contact angle measurements for the 3M 1860 N95 FFR material, both the dry heat and steam treatments show an increase in the observed contact angle in comparison to that of the pristine, which evince an initial contact angle of 103.9°±7.7°, 105.4°±6.2°, and 96.0°±15.2°, respectively. The treated samples’ contact angle values remain consistent over time, whereas the pristine sample showed a marked decrease. No significant difference is shown between the droplet volume over time for the three samples. This observation suggests that the wettability of the pristine sample increases over time, but this behavior is ameliorated by the dry heat and steam treatments. For all three samples, the inner surface rate of absorption was too rapid to allow for measurements by contact angle with the 20 μl droplet being absorbed during the first 1000 μs measurement interval. (TIFF) [file pone.0257963.s003.tiff]

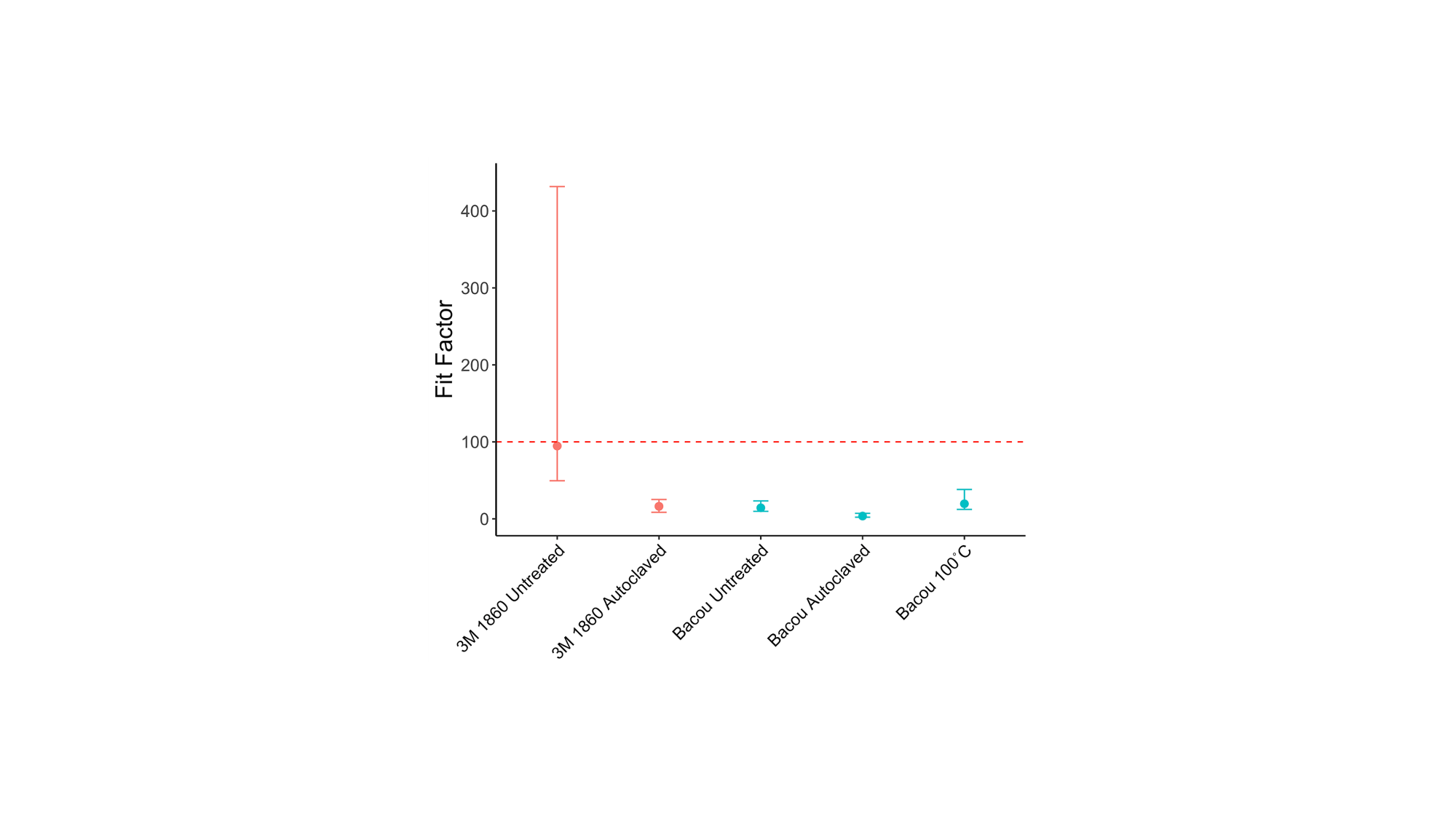

Supplement: S3 Fig — We derived the fit factor as defined in OSHA guidelines (see Methods section). Upper and lower bounds of the fit factor assumed the most conservative count estimates applying measured counts and their corresponding count error. Most conservative signifies, e.g., the greatest number of 40 nm particle in room air (including count uncertainty) over lowest number of 40 nm particles in respirator (subtracting count uncertainty). (TIFF) [file pone.0257963.s004.tiff]

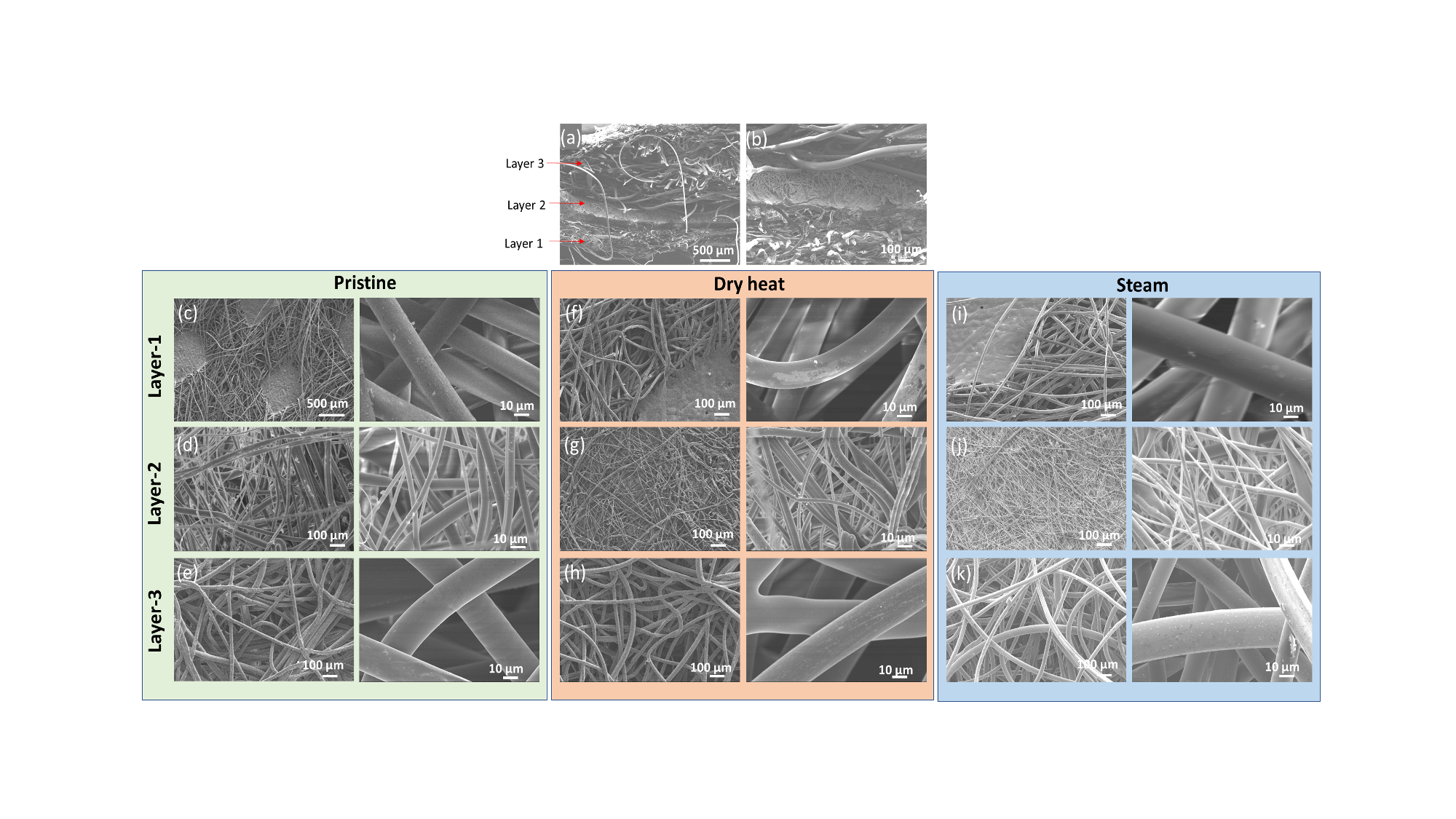

Supplement: S4 Fig — (a-b) SEM images of cross section. (c-e) SEM images of top down view of pristine Bacou Willson 801 N95 FFR. (f-h) SEM images of top down view of the of Bacou Willson 801 N95 after heat treatment at 100°C for 4 cycles. (i-k) SEM images of top down view of the of Bacou Willson 801 N95 after steam treatment. The morphologies of dry air heat treatment and steam (autoclave) treatment do not show obvious difference from SEM images, which indicates the morphologies are not changed under the dry air heat treatment and steam treatment methods used. (TIFF) [file pone.0257963.s005.tiff]

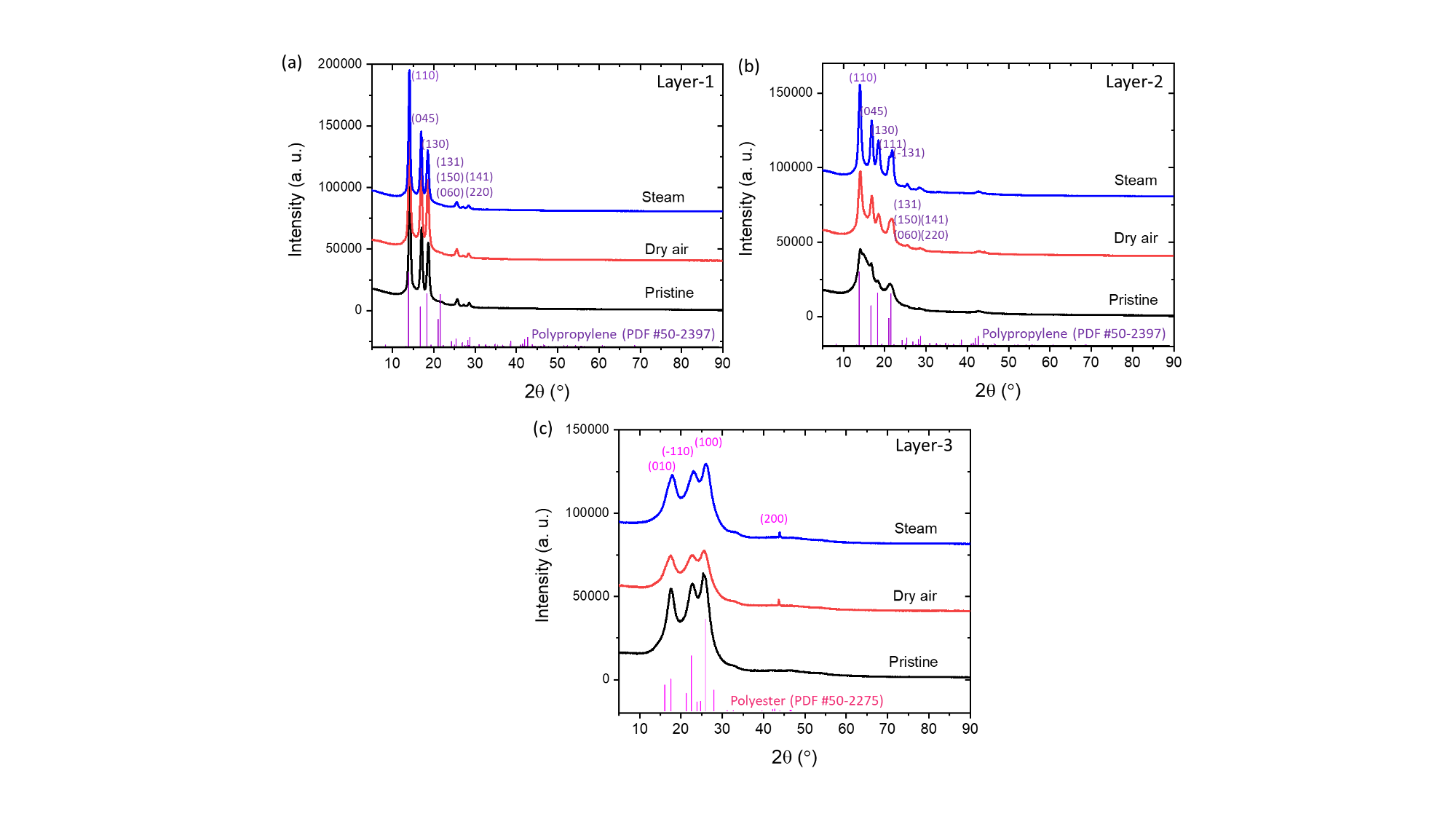

Supplement: S5 Fig — XRD of three layers in Bacou Willson 801 N95 (a-c) before and after dry heat and steam heat treatment. The XRD patterns of layer 1 and layer 2 indicated a semicrystalline character, and as marked therein, the major diffraction patterns were indexed to reflections from (110), (045), (130) and (-131) planes of the polypropylene phase (PDF #50–2397). Layer 1 showed larger crystallite size (16 nm) than layer 2 (4 nm), indicating layer 1 is more crystalline than layer 2 in the pristine mask (S5a-b, black curves). However, in layer-2 an extra peak at 2θ = 20.07֯ was evident (S5b), corresponding to the (111) peak of polypropylene. The XRD patterns of the layer-3 also indicated a semicrystalline character, and as marked therein, the major diffraction patterns were indexed to reflections from the (010), (-110) and (100) planes of the polyester phase (PDF #50–2275) (S5c). Compared with the XRD patterns of pristine (untreated) Bacou Willson 801 N95 (black curve), the XRD patterns after dry air treatment (red) and steam treatment (blue) indicate higher crystallinity for layers 1 and 2 (S5-b). Specifically, the crystallite sizes of the respective pristine, dry heat treated, and steam treated 16, 18 and 19 nm for layer 1 (S5a) and 4, 8 and 12 nm for layer 2 (S5a). This indicated that steam treatment increases crystallite size more than dry air treatment at both layer 1 and layer 2, and the dry air treatment and steam treatment has more effect on crystallinity of layer 2 than layer 1. Layer 3 is more amorphous with significant peak overlap, so no crystallite size was calculated on layer 3, but an extra peak at 2θ = 48.36 were observed after dry air and steam treatment, which corresponds to the (200) peak of polyester. (S5c). (TIFF) [file pone.0257963.s006.tiff]

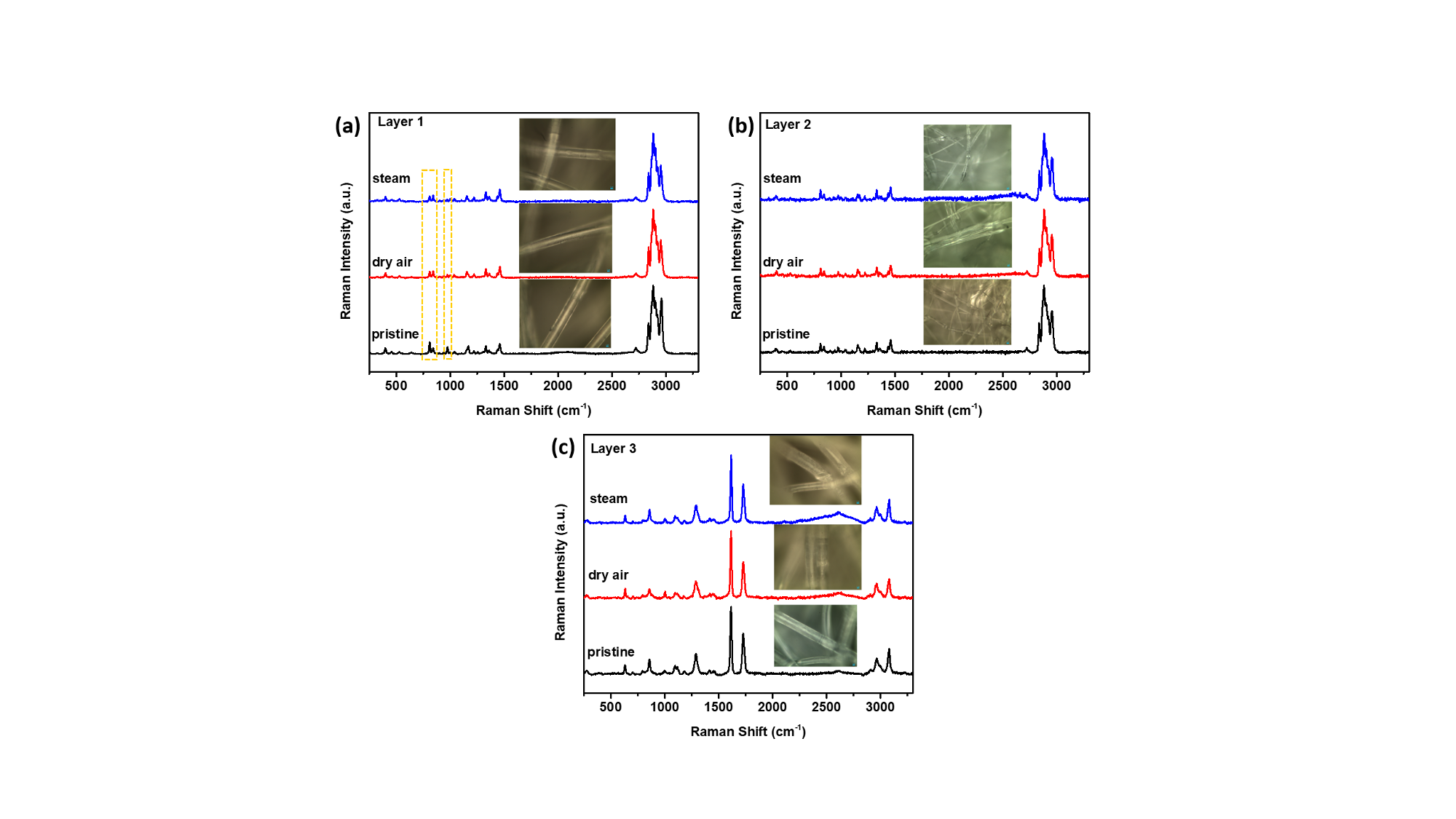

Supplement: S6 Fig — Based on the acquired Raman spectra, layer 1 and 2 of Bacou Willson 801 N95 FFR (S6a & c) have spectra features resembling polypropylene materials. After the dry air and steam treatment, the ratio of the two bands at 810 and 840 cm-1 layer 1 decreased, along with the decreasing intensity of the 972 cm-1 peak (S6a), as highlighted in the yellow dashed regions, suggesting a shorting of the helical chain conformation of polypropylene after heat treatment [28]. The regularity bands at 973, 998, 841, and 1220 cm–1 were previously assigned to the helical chains of 5, 10, 12, 14 monomeric units of polypropylene, respectively. The 2nd layer of the Bacou Willson 801 N95 FFR material (S6) showed broader and weaker peaks than those in layer 1, which potentially suggested lower crystallinity in this layer consistent with the narrower thickness of the fibers [34]. No significant changes were noted after the heat treatment in this layer. The 3rd layer of the Bacou Willson 801 N95 FFR can be assigned to polyester (S6c),[35] as indicated by the strong C = C stretching band (ring deformation) at 1615 cm-1 and C = O stretching band at 1730 cm-1. Similarly, no significant differences in Raman spectra were observed in the bulk structure of layer 3 before and after heat treatment, suggesting minimal changes in crystallinity and bond orientation implying that layers 2 and 3 of Bacou Willson 801 N95 FFR are stable under heat treatment. (TIFF) [file pone.0257963.s007.tiff]

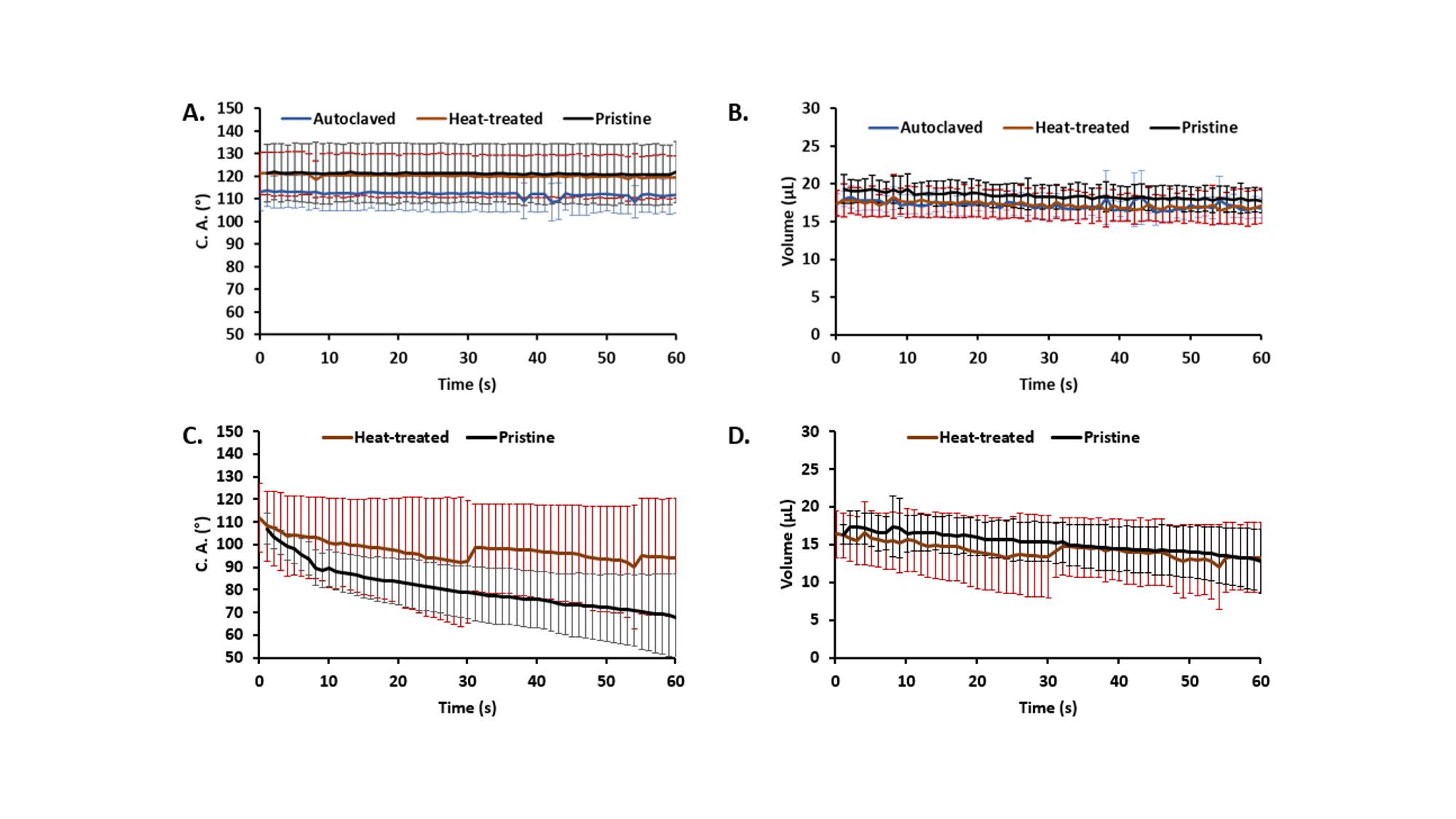

Supplement: S7 Fig — Contact angle and droplet volume over time of outer (a & b) and inner (c & d) surfaces of Bacou Willson 801 N95 FFR before and after dry heat and steam treatment. There is not an observed significant difference between the contact angle of the pristine, dry heat treated, and steam treated Bacou Willson 801 N95 FFR samples, which evince an initial contact angle of 121.4°±12.6°, 121.2°±9.5°, and 113.1°±8.3°, respectively, and remain constant over time. Though the steam treated (autoclaved) sample shows the greatest reduction in contact angle, suggesting an increase in surface adsorption of the artificial saliva, this value still lies within the error of the pristine (untreated) measurement. Furthermore, the same similarity in surface absorption is shown by the observation of the volume of the liquid droplet over time. Greater variability is observed when measurements are taken of the inner surfaces of the Bacou Willson 801 N95 FFR samples, (S7c & d). Whilst there are no significant initial differences between the pristine and heat-treated samples, 106.9°±6.8° and 111.9°±15.1°, respectively, both samples show a decreasing trend over time. The most rapid decrease is observed within the pristine sample, which achieves a value of 64.4°±15.2° after 1 minute. This increase in surface adsorption and wettability of the inner surfaces is further supported by the increase rate of surface absorption suggest by negative slope of the volume over time figure. The steam treated sample rate of surface absorption was too rapid to allow for measurements by contact angle with the 20 μl droplet being absorbed with first 1000 μs measurement interval. The wetting properties of the inner mask to outer mask surfaces suggest the inner surfaces draw respiratory expulsions away from the user whereas the outer surfaces can repel respiratory expulsions toward the user from other sources. Dry heat treatments decrease the absorption of the inner layer; however, steam treatments induce a distinct in [file pone.0257963.s008.tiff]
