# Supplementary material for: Dry heat sterilization as a method to recycle N95 respirator masks: The importance of fit
Source: PLoS One. 2022 Jan 5;17(1):e0257963. doi: 10.1371/journal.pone.0257963 (PMC8730429; doi:10.1371/journal.pone.0257963)
Supplement: Attachment — Submitted filename: Response to Reviewers.docx [file pone.0257963.s009.docx]

Manuscript PONE-D-21-27839

Response to Reviewers

Dear Dr. Singh and Reviewers,

Thank you for the feedback and for giving us the opportunity to submit a revised draft of the manuscript “Dry heat sterilization as a method to recycle N95 respirator masks: the importance of fit” for publication in PLOS ONE. We appreciate the time and effort that you and the reviewers dedicated to the review of the manuscript, and we are grateful for the insightful comments. We have incorporated the suggestions made by the reviewers and have outlined them below and within the manuscript.

**Reviewer #1:**

The manuscript is technically sound and well written. However, in the results section the complete data for thermal stability on N95 Respirators seems to be missing. Thus same can be provided in the supporting information.

**Author response:** Thank you very much! As for the data for thermal stability, the experiments were done in triplicate, where SARS-CoV-2 virus was recovered by cutting each virus-treated spot, from each N95 mask and submerging the samples in media. Briefly, samples were incubated for 5 minutes at room temperature then rocked gently to recover virus. Plaque assays were performed to quantify the amount of virus recovered by performing serial dilutions of recovered virus and infecting VeroE6 cells for one hour. To quantify the amount of virus recovered, the overlay was removed, and the plaques were visualized by staining VeroE6 cells with 0.5% crystal violet and 0.8% glutaraldehyde in 50% methanol. Total PFU/mL for each condition was calculated by averaging the mean PFU/mL recovered for each biological replicate. Additional details, including the description above, are written in the Methods section “SARS-CoV-2 Thermal Stability/Viral Recovery and Quantification.”

Comment 1: Introduction section line 83; “…OSHA compliant fit factors that quantitatively evaluate whether a respirator fits properly…”. OSHA is used for the first time Thus it is advised to write the full form of OSHA over here.

**Author response:** Thank you for pointing this out, we have written out “Occupational Safety and Health Administration” as advised in line 84 of the revised manuscript.

Comment 2: Materials and Methods section line 123; “VeroE6 cells were routinely cultured in DMEM containing Glutagro (Corning) and 8%...”. writing the full form of DMEM media is recommended.

**Author response:** Thank you for pointing this out, we have written out “Dulbecco’s Modified Eagle Medium” as advised in line 140 of the revised manuscript.

Comment 3: N95 FFR Quantitative Fit Tests line 157-158; “Quantitative fit testing was performed on the same operator for all mask types and for all conditions:

dry heat treated (n = 3), untreated (n = 1), and autoclaved (n = 1) respirators”. As comparison is done between dry heat treated, untreated, and autoclaved respirators taking the value of n 3, 1, and 1 respectively, then why the heterogeneity is there in terms of the sample size n?

**Author response:** Thank you for pointing this out. At the time the experiment was conducted, there was a shortage of N95 respirators, and the supply of respirators were tightly regulated by Environmental Health and Safety at our institution. We therefore decided to take a conservative approach, as we must destroy the N95 respirators in order to perform fit testing by puncturing the masks in order to affix a valve onto the respirator. Similarly, autoclaving a N95 respirator will undoubtedly damage the respirator as reported in the literature (doi: [10.1016/j.jhin.2020.06.030](https://dx.doi.org/10.1016%2Fj.jhin.2020.06.030), [10.1371/journal.pone.0243965](https://doi.org/10.1371/journal.pone.0243965)) and as observed in our experiment by the deformation of the respirator.

Comment 4: N95 FFR Quantitative Fit Tests line 159-165; “Quantitative fit testing procedures for N95 respirators were performed according to Occupational Safety…. passes OSHA guidelines”. It would be preferable for the readers if the authors could add the reference in this paragraph.

**Author response:** Thank you for pointing this out. We have added the appropriate citation.

Comment 5: N95 FFR Quantitative Fit Tests line 166. Please check the formula of the Overall Fit factor and correct the running variable (or summation index). Also, it is advised to the author to describe the term n in the text as this will help the reader to understand better.

**Author response:** Thank you for pointing this out. We have clarified the definition of n in the text as the number of exercises and corrected the typo in the summation index.

Comment 6: N95 FFR Quantitative Fit Tests line 168. It would be preferable for the readers if the authors could add the reference for Table 1.

**Author response:** Thank you for pointing this out. We have added the citation to Table 1.

Comment 7: Estimated Filtration line 285-286. “A fit factor of 100 and 200 corresponds to a filtration efficiency of 99 and 99.5% respectively (both imply passing of the fit test)”. How this relation between fit factor and filtration efficiency is drawn? It would be preferable for the readers if the authors could offer additional detail and explain the reason behind this trend.

**Author response:** Thank you for pointing this out. We have clarified that the estimated filtration can be calculated based off the definition of a fit factor (the ratio of the concentration of ambient particles to the concentration of particles inside the respirator), thus is equal to 1 – 1/FitFactor. These changes are reflected in lines 349-351.

Comment 8: Estimated Filtration line 290. In table 2 there is no indication of the calculation used to determine estimated filtration (%). Thus, it would be preferable for the readers if the authors could include the information of calculation done.

**Author response:** We think this is an excellent suggestion and have addressed it with the same clarifications we made in response to Comment 7.

Comment 9: Please check the uniformity of the References. For instance: Few details of reference 11 are missing.

**Author response:** Thank you for pointing this out. We have double checked the references and added the missing details to references 11 and 2.

**Reviewer #2:** The work submitted is interesting and can be accepted with minor revisions.

**Author response:** Thank you very much!

Please check the notations throughout the manuscript and keep it uniform.

**Author response:** Thank you for pointing this out. We have made some revisions on the notation and have clarified the usage of mask, respirator, and FFR according to their most precise and accurate definitions as most commonly used by the CDC, NIOSH, OSHA and the manufacturers.

Line 71: Please mention the time as it is a critical parameter for autoclaving

**Author response:** Thank you for pointing this out. The parameters are detailed more thoroughly in the Methods section, where we have stated that the autoclave time is for 30 minutes. The mention of autoclave conditions in line 71 are to point out in the Introduction that autoclaving involves both steam and pressure and typical conditions are given.

Follow the units in the same format throughout

**Author response:** Thank you for pointing this out, we have double checked the units and have made revisions in the text.

Please provide a reference for Table 1 with a source

**Author response:** Thank you for pointing this out. We have added the appropriate citation.

Please improve figure one

**Author response:** Thank you for the suggestion. We have decided that this figure does not add much to the manuscript, and we have therefore removed it.
